# Biophysical Property and Broad Anti-HIV Activity of Albuvirtide, a 3-Maleimimidopropionic Acid-Modified Peptide Fusion Inhibitor

**DOI:** 10.1371/journal.pone.0032599

**Published:** 2012-03-05

**Authors:** Huihui Chong, Xue Yao, Chao Zhang, Lifeng Cai, Sheng Cui, Youchun Wang, Yuxian He

**Affiliations:** 1 Institute of Pathogen Biology, Chinese Academy of Medical Sciences and Peking Union Medical College, Beijing, China; 2 Beijing Institute of Pharmacology and Toxicology, Beijing, China; 3 National Institute for the Control of Pharmaceutical and Biological Products, Beijing, China; Scripps Research Institute, United States of America

## Abstract

Albuvirtide (ABT) is a 3-maleimimidopropionic acid (MPA)-modified peptide HIV fusion inhibitor that can irreversibly conjugate to serum albumin. Previous studies demonstrated its *in vivo* long half-life and potent anti-HIV activity. Here, we focused to characterize its biophysical properties and evaluate its antiviral spectrum. In contrast to T20 (Enfuvirtide, Fuzeon), ABT was able to form a stable α-helical conformation with the target sequence and block the fusion-active six-helix bundle (6-HB) formation in a dominant-negative manner. It efficiently inhibited HIV-1 Env-mediated cell membrane fusion and virus entry. A large panel of 42 HIV-1 pseudoviruses with different genotypes were constructed and used for the antiviral evaluation. The results showed that ABT had potent inhibitory activity against the subtypes A, B and C that predominate the worldwide AIDS epidemics, and subtype B′, CRF07_BC and CRF01_AE recombinants that are currently circulating in China. Furthermore, ABT was also highly effective against HIV-1 variants resistant to T20. Taken together, our data indicate that the chemically modified peptide ABT can serve as an ideal HIV-1 fusion inhibitor.

## Introduction

HIV-1 envelope glycoprotein (Env) complex, which is composed of three receptor-binding gp120 subunits and three fusion protein gp41 subunits, mediates virus entry by fusing viral and cellular membranes and offers an attractive target for developing antiviral agents. During the fusion reaction, N- and C-terminal heptad repeat regions (NHR and CHR) of the gp41 ectodomain refold into a thermostable six-helix bundle structure (6-HB), representing a fusion-active conformation that can bridge the viral and cellular membranes for the merger [1]–[4]. A number of peptides derived from the gp41 CHR (C-peptides) can specifically inhibit viral entry at low nanomolar concentration, such as C34, T20 [5]–[9]. It is thought that C-peptides act by competitive binding to the NHR of gp41 during its conformational change to the fusogenic state (i.e. pre-hairpin conformation) and hence block the 6-HB formation in a dominant-negative manner [6]–[7], [10]. T20 (Enfuvirtide, Fuzeon) has been successfully developed as the first and only HIV-1 fusion inhibitor for clinical use [11]–[12]. However, peptide drugs usually suffer from their short *in vivo* half-life and require frequent injections. Being a 36-mer peptide, T20 has an *in vivo* half-life of 3.46 to 4.35 h and is given twice-daily at a high-dosage [13]. Therefore, development of long-acting anti-HIV peptide drugs is highly intriguing.

In succession to T20, a number of design strategies have been applied to develop new peptide-based fusion inhibitors with improved stability, bioavailability and potency [5]–[7], [14]–[18]. For example, several potent inhibitors were designed by introducing intra-helical salt-bridges that can stabilize the peptides [16]–[17], [19]. T2635, a third generation peptide inhibitor is highly active against HIV-1 escape variants [17]–[18]. It is believed that the charged residues within T2635 are “masked” by introduced salt bridges thus overcoming the resistance by mechanism of charge-repulsion [14], [17], [20]. Another successful example is Sifuvirtide (SFT), an electrostatically constrained peptide inhibitor showing potent anti-HIV activity, good safety and pharmacokinetic profiles, and is currently under Phase II clinical trials [16], [21]. Recently, peptide-fatty acid and cholesterol conjugates with dramatically improved inhibitory activity and pharmacokinetics have been reported [22]–[23]. It was hypothesized that the incorporation of fatty acid or cholesterol can target the peptide inhibitors to viral or cellular membranes thereby increasing the drug concentration at the site of membrane fusion. Prominently, the cholesterol-conjugated peptide C34 had dramatically increased serum lifetime in mice [23]. Stoddart *et al* [24] reported that human serum albumin (HSA)-conjugated peptides (T20 and C34) possessed equipotent *in vitro* anti-HIV activity compared to the original peptides and sustained activity *in vivo*.

**Figure 1:**
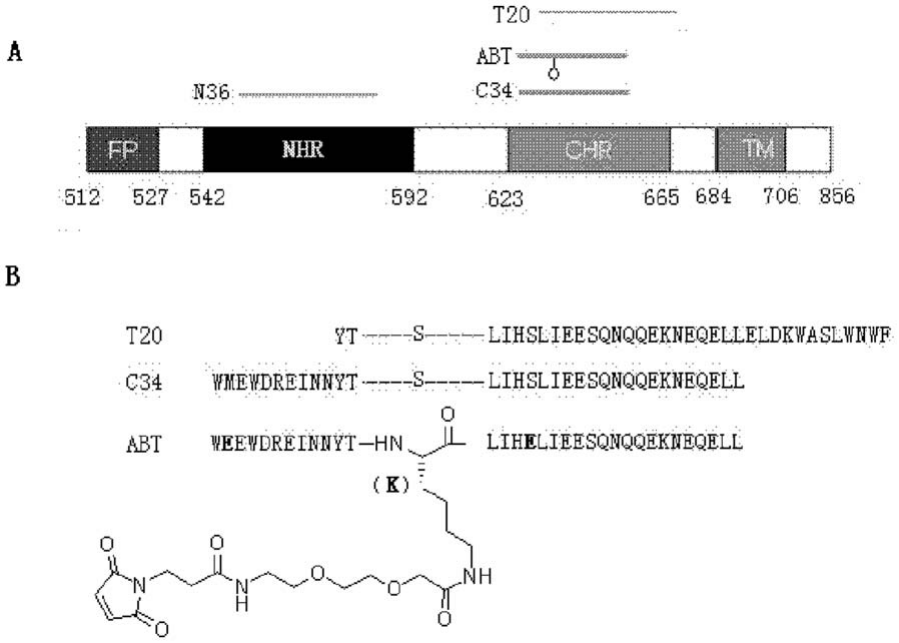
Schematic illustration of HIV-1 gp41 and peptide fusion inhibitors. A. View of the gp41 functional regions. The residue numbers of each region correspond to their positions in gp160 of HIV-1_HXB2_. FP, fusion peptide; NHR, N-terminal heptad repeat; CHR, C-terminal heptad repeat; TM, transmembrane domain. B. Sequence of CHR-derived anti-HIV-1 peptides. ABT is engineered with three amino acids different from C34 (marked in bold). The 13th residue serine (S) of C34 was changed to lysine (K) which allows a single modification by 3-maleimidopropionic acid (MPA).

Albuvirtide (ABT) is a 3-maleimimidopropionic acid (MPA)-modified peptide designed with C34 sequence as a template (Fig. 1) [25]. In which, the 13th residue serine (S) was changed to lysine (K) which allows a single MPA modification at this position. Other two residues not involved in target binding were substituted by glutamic acid (E) to improve the solubility, stability and antiviral activity. The engineered peptide can covalently link to serum albumin at a 1∶1 molecular ratio through an irreversible conjugation reaction between the maleimide and free thiol groups [25]. Previous studies demonstrated that upon intravenous injection ABT could rapidly form conjugates with serum albumin and dramatically extend its *in vivo* half-life from 1.67 h to 25.8 h in rats and from 10.89 h to 102.4 h in monkeys [25]. Importantly, ABT had potent *in vivo* anti-HIV efficacy as shown by a SCID-huThy/Liv mouse infection model, highlighting its potential to become a new generation HIV-1 fusion inhibitor. A Phase I clinical trial is under way to evaluate its safety, tolerability and pharmacokinetic profiles in humans. In this parallel leading study, we are interested in characterizing its biophysical properties and antiviral spectrum. Our results demonstrated that the chemically modified ABT could form a stable helical structure with the target sequence and efficiently block 6-HB formation and HIV-1 Env-mediated cell-cell fusion. Prominently, it inhibited viral entry by diverse HIV-1 subtypes and variants, including the subtypes A, B and C that predominate the worldwide AIDS epidemics, and subtype B′, CRF07_BC and CRF01_AE recombinants that are currently circulating in China, and HIV-1 variants resistant to T20. The data provide critical information for developing new HIV-1 fusion inhibitors for clinical use.

## Results

### Biophysical characterization of ABT

**Figure 2:**
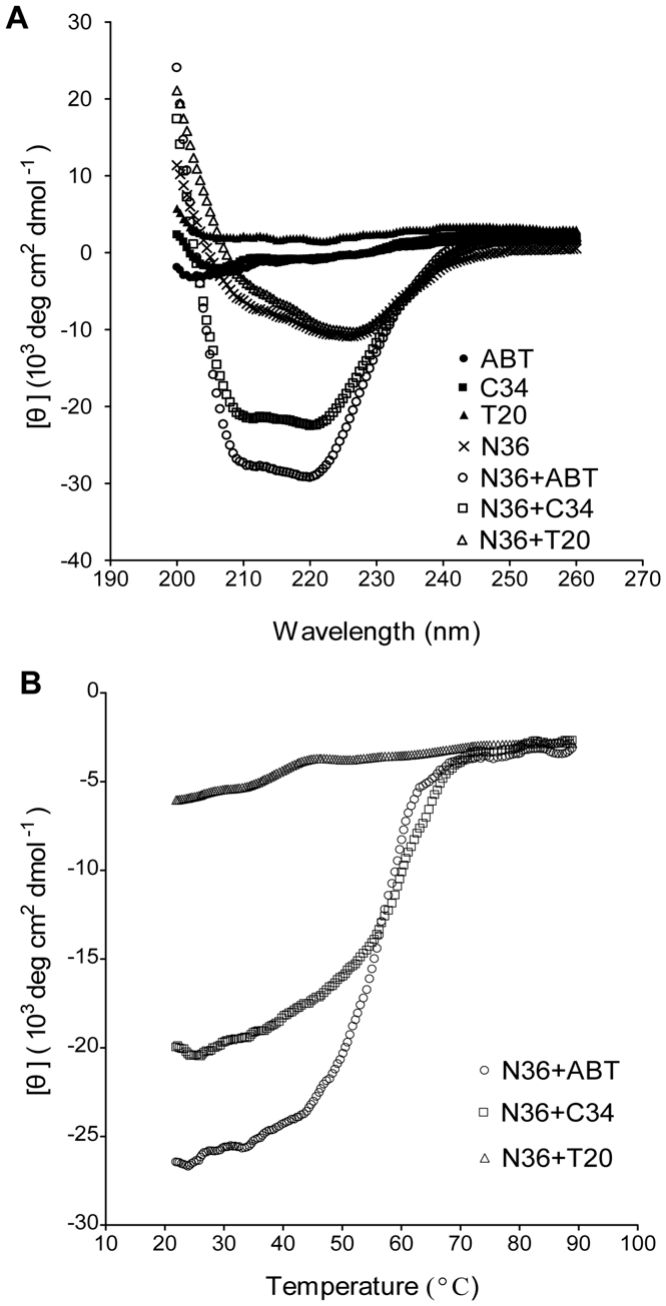
Biophysical characterization of ABT by CD spectroscopy. A. CD spectra of NHR and CHR-derived peptides and their complexes. B. Thermostability of the complex formed by N36 and ABT or C34. The unfolding temperature of each complex was scanned at 222 nm by CD spectroscopy, and their *Tm* values were calculated. The final concentration of each peptide in PBS is 1 µM.

To clarify whether the MPA modification affects the secondary structure of ABT, we examined its interaction with the NHR target sequence (N36) using CD spectroscopy. C34 and T20 were included as control peptides. As shown in Fig. 2, the CD spectra of C-peptides alone (ABT, T20 and C34) did not show a minimum at 222 nm, suggesting their minimum α-helical conformation. In the presence of N36, the CD spectra of both ABT and C34 displayed a double minimum at 208 and 222 nm, suggesting that the interactions of C- and N-peptides induced the formation of secondary α-helical structures. Apparently, the ABT/N36 complex had a relatively higher α-helicity than C34/N36 complex. Consistent with our previous studies, T20 could not form a typical α-helical complex with N36 as indicated by the lack of minimum at 208 and 222 nm. To determine the binding affinity of ABT to N36, we measured the thermal stability of the ABT/N36 helical complex. The sigmoidal transition of the CD signal at 222 nm correlates with the thermal stability of the helical complexes formed from C- and N-peptides, which in turn reflects the binding affinity of these peptides. Fig. 2B shows the CD thermal denaturation curves of C-peptides and N36 complexes. Compared to the C34/N36 (*Tm* = 54°C), the ABT/N36 had a slightly improved thermal stability (*Tm* = 56°C) while the T20/N36 mixture was undetectable.

**Figure 3:**
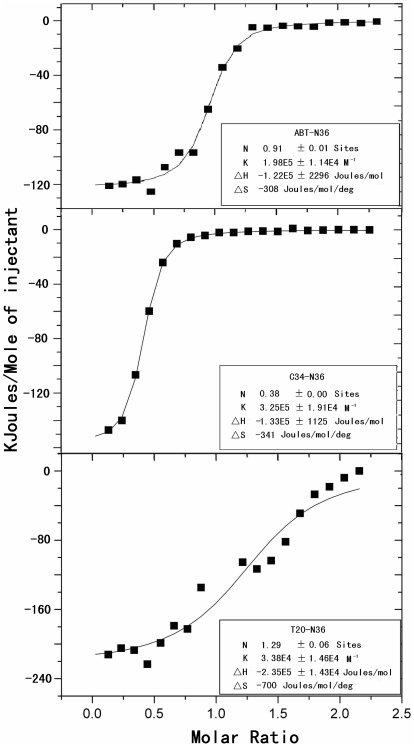
Biophysical characterization of ABT by ITC assay. 2 mM N36 dissolved in ddH_2_0 was injected into the chamber containing 200 µM ABT, C34 or T20. The experiments were carried out at 25°C. Data acquisition and analysis were performed using MicroCal Origin software (version 7.0), and show that ABT and C34 can efficiently interact with N36 but T20 not.

Further, we used an ITC assay to determine the interaction between C-peptides and N36. Consistently, both ABT and C34 could specifically interact with N36 in our experimental conditions (Fig. 3). In contrast, T20 could not efficiently interact with N36 as manifested by its low Kd value compared to ABT and C34. Such biophysical characteristics of ABT indicate that this MPA-modified peptide maintains its normal structure to form α-helical interactions with the N-helices.

### Inhibition of ABT on 6-HB formation, cell fusion, viral entry and replication

**Figure 4:**
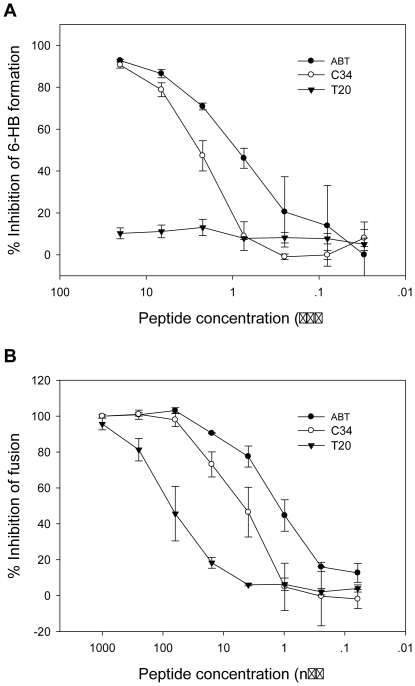
Inhibition of ABT on 6-HB formation and cell membrane fusion. A. ABT and C34 can efficiently inhibit 6-HB formation in a dose-dependent manner, but T20 has no such effect. B. Inhibition of HIV-1_HXB2_ Env-mediated cell-cell membrane fusion by ABT, C34 and T20. The data were derived from the results of three independent experiments and are expressed as means ± standard deviations.

To elucidate the mechanism of action of ABT, we checked whether ABT can physically block the formation of 6-HB as modeled by the N36 and C34 peptides described previously [16], [26]. As shown in Fig. 4A, both ABT and C34 were able to inhibit the formation of 6-HB in a dose-dependent fashion, but the former exhibited significantly higher potency than C34 as manifested by its IC_50_ value of 0.82 µM compared with 3.25 µM of C34. Unlike ABT and C34, T20 had no such effect at a concentration as high as 20 µM. These results were consistent with our observations from CD studies and demonstrated that ABT acts in a mechanism of blocking 6-HB formation in a dominant-negative manner. Further, we examined the inhibitory activity of ABT on HIV-1_HXB2_ Env-mediated cell-cell membrane fusion. As shown in Fig. 4B, ABT could inhibit the fusion with an IC_50_ of 1.27 nM, while C34 and T20 inhibited the fusion with IC_50_ values of 3.86 nM and 56.22 nM, respectively.

**Figure 5:**
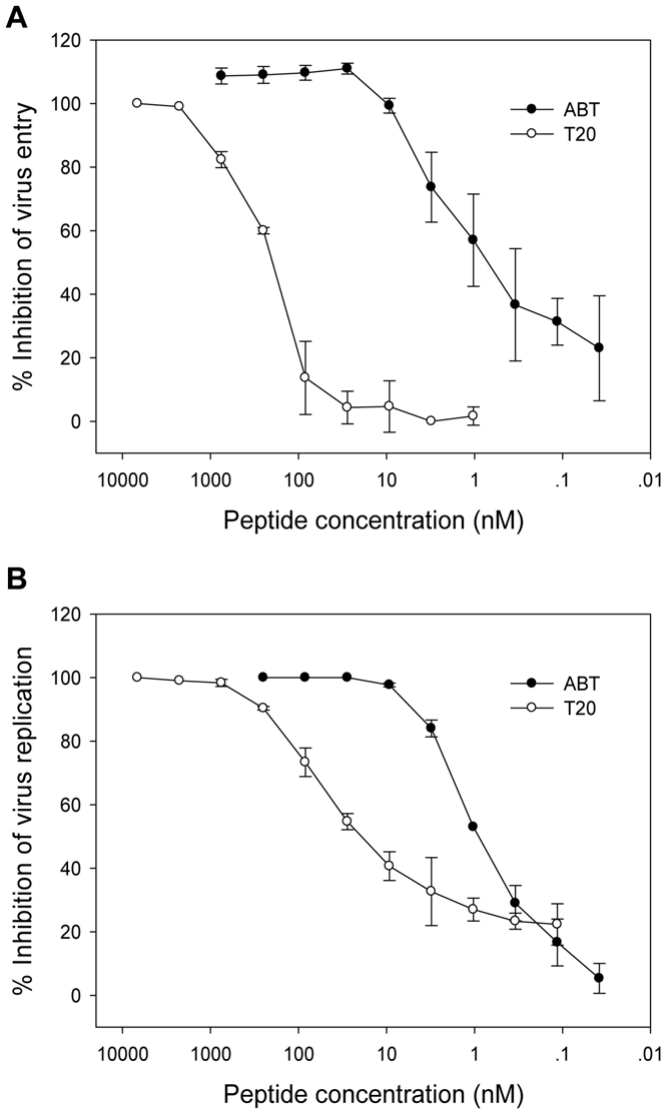
Inhibition of ABT on HIV-1 entry and replication. A. Inhibition of HIV-1_NL4-3_ Env-pseudotyped virus in single-cycle assay that demonstrates the virus-cell membrane fusion. B. Inhibition of wild-type HIV-1_NL4-3_ replication. ABT shows significantly higher potency than T20 in inhibiting HIV-1_NL4-3_ entry and replication. The data were derived from the results of three independent experiments and are expressed as means ± standard deviations.

**Figure 6:**
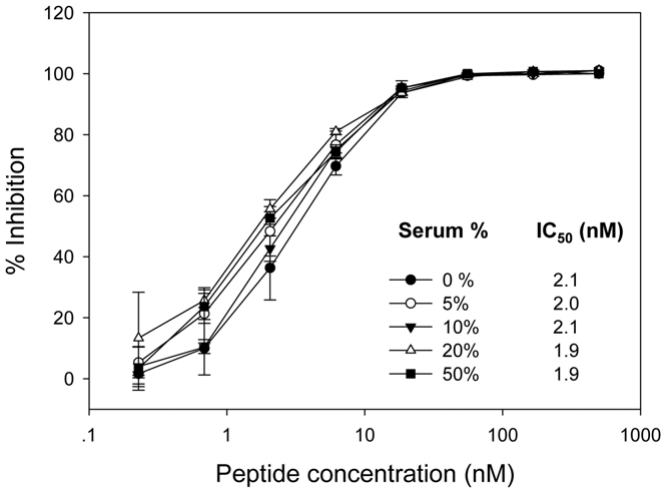
Effect of human serum on the anti-HIV activity of ABT. The human sera were freshly isolated from a HIV-seronegative healthy volunteer. The peptide was mixed with various concentrations (5, 10, 20 and 50%) of human sera freshly isolated from a HIV-seronegative healthy volunteer and incubated for 2 h at 37°C. The mixture was then diluted with a DMEM-based complete medium supplemented with 10% FCS and subjected to the single-cycle infection assay. The percent inhibition by ABT was calculated as described in the materials and method. The data were derived from the results of three independent experiments and are expressed as means ± standard deviations.

Next, we tested ABT and T20 for their inhibitory activity on the viral cell entry mediated by HIV-1_NL4-3_ Env-pseudotyped virus. As shown in Fig. 5A, ABT could efficiently inhibit the virus entry with a mean IC_50_ at 2.19 nM, whereas T20 worked with much less efficiency (IC_50_ = 70.74 nM). Similarly, ABT had much potent inhibitory activity on the replication of wild-type HIV-1_NL4-3_ (Fig. 5B). In addition, we assessed ABT for its antiviral activity in the presence of serum components. The peptide was mixed with various concentrations (5, 10, 20 and 50%) of human sera freshly isolated from a HIV-seronegative healthy volunteer and incubated for 2 h at 37°C. The mixture was then diluted with a DMEM-based complete medium supplemented with 10% FCS and subjected to the single-cycle infection assay. From Fig. 6 we can see that the HIV-inhibitory activity of ABT was not influenced by high concentration of human sera (up to 50%).

### Potent inhibition of ABT on HIV-1 subtypes that dominate the AIDS epidemics worldwide

HIV-1 evolves with the great genetic diversity and can be classified into three major groups: M (major), O (outlier) and N (non-M non-O or new) [27]–[28]. The M group viruses that cause the vast majority of HIV-1 pandemic can be further divided into many subtypes, including A–D, F–H, J and K, as well as several circulating and unique recombinant forms (CRFs and URFs). The sequence diversity has raised concerns over the peptide fusion inhibitors that target the highly variable envelope region. Here, we firstly evaluated ABT for its antiviral activity with a panel of 14 HIV-1 pseudoviruses with their *Envs* derived from subtypes A, B and C. As shown in Table 1, ABT had potent inhibitory activity against the cellular entry by these diverse viruses. It inhibited subtype A, B and C viruses with mean IC_50_ values at 6.3 nM, 27.41 nM and 2.92 nM respectively. In comparison, T20 showed much weak inhibition on these three subtypes, with mean IC_50_ at 14.47 nM, 214.04 and 55.18 nM respectively. Compared to other HIV-1 isolates, some Envs derived from the subtype B were much less sensitive to both ABT and T20, but sequence analysis did not identify the previously known resistance mutations in the NHR and CHR of gp41.

**Table 1:** Inhibition of ABT on subtype A, B and C HIV-1 strains^a^.

Pseudovirus	Subtype	CoR^b^	IC50 (nM)	
			ABT	T20
92RW020	A	R5	5.65±2.17	25.38±9.81
92UG037.8	A	R5	6.95±0.66	3.55±0.95
SF162.LS	B	R5	12.79±1.18	43.55±9.16
AC10.0.29	B	R5	5.64±1.20	10.07±0.45
TRO.11	B	R5	35.10±6.01	333.65±48.86
REJO4541	B	R5	3.12±0.17	78.07±11.26
RHPA4259	B	R5	66.39±4.47	281.90±0.57
CAAN5342	B	R5	63.05±1.64	722.90±53.46
SC422661.8	B	R5	5.81±0.51	28.17±5.92
Du172.17	C	R5	1.01±0.39	11.60±5.29
Du422.1	C	R5	1.46±0.26	28.42±5.14
ZM197M.PB7	C	R5	5.42±0.71	63.30±17.01
ZM109F.PB4	C	R5	2.89±0.78	76.38±2.69
CAP45.2.00.G3	C	R5	3.83±0.19	96.22±6.91

a The inhibitory activity of each peptide was determined in triplicate by a single-cycle infectivity assay. The data were derived from the results of three independent experiments and are expressed as means ± standard deviations.

b Co-R: coreceptor use.

### Potent inhibition of ABT on HIV-1 variants that are predominantly circulating in China

Because ABT has been approved for Phase I clinical trials, we are interested to know the inhibitory activity of ABT on HIV-1 strains that are currently circulating in China. Two recombinant forms, CRF07_BC and CRF01_AE, and subtype B′ are predominant viruses, accounting for infections at 50.2%, 15.54% and 29.11% respectively (with a total of ∼95%) [29]–[30]. Therefore, we constructed a panel of 28 HIV-1 pseudoviruses with their Envs derived from the above three subtypes and used in single-cycle infection assays. The results have been presented in Table 2. Compared to T20, ABT displayed more potent inhibitory activity against the virus entry, with mean IC_50_ at 5.21 nM for CRF07_BC, 6.93 nM for CRF01_AE, and 9.46 nM for B′. T20 inhibited three subtypes at 47.2 nM, 68.11 nM and 19.49 nM respectively.

**Table 2:** Inhibition of ABT on CRF07_BC, CRF01_AE and B'HIV-1 variants^a^.

Pseudovirus	Subtype^b^	CoR^c^	IC50 (nM)	
			ABT	T20
CH064	B/C	R5	2.33±0.21	61.72±12.54
CH070	B/C	R5	6.23±0.63	135.95±36.42
CH091	B/C	R5	2.82±0.35	65.93±14.28
CH110	B/C	R5	2.80±0.61	18.57±3.15
CH119	B/C	R5	6.71±1.15	22.11±2.74
CH120	B/C	R5	16.00±1.68	50.43±4.22
BC02	B/C	R5	1.51±0.13	2.60±0.48
BC03	B/C	R5	3.51±0.50	3.46±0.41
BC05	B/C	R5	5.92±0.26	4.39±0.25
BC07	B/C	R5	5.46±1.03	12.52±1.58
BC14	B/C	R5	3.43±0.04	108.51±52.63
BJ22-5	B/C	R5	4.10±0.22	25.78±4.17
HB5-3	B/C	R5	2.70±0.40	23.11±2.55
SC19-15	B/C	R5	1.31±0.10	7.61±0.97
YN148r-9	B/C	R5	16.88±1.62	131.43±14.74
XJ50-6	B/C	R5	1.59±0.17	81.08±17.61
AE01	A/E	R5	2.26±1.01	24.83±2.25
AE03	A/E	R5	7.79±0.27	7.80±1.66
BJ5.11	A/E	R5	17.09±1.85	342.00±132.09
SHX335.24	A/E	R5	1.33±0.34	51.00±7.35
YN192.31	A/E	R5	11.41±1.84	68.53±3.25
GX11.13	A/E	R5	5.78±1.00	6.60±0.32
GX2010.36	A/E	R5	4.36±0.33	18.14±3.75
GX2010.36H	A/E	R5	5.43±0.45	25.94±0.28
B01	B′	R5	2.19±0.10	49.50±2.41
B02	B′	R5	9.25±1.00	8.59±0.80
B04	B′	R5	18.10±5.30	7.80±0.43
43-22	B′	R5	8.28±0.85	12.07±3.24

a The inhibitory activity of each peptide was determined in triplicate by a single-cycle infectivity assay. The data were derived from the results of at least three independent experiments and are expressed as means ± standard deviations.

b HIV-1 subtypes: B/C, CRF07_BC; A/E, CRF01_AE; B′, Tai B.

c Co-R: coreceptor use.

### Potent inhibition of ABT on HIV-1 carrying mutations that confer T20-resistance

Because there were significant differences in biophysical and anti-HIV properties between ABT and T20, we sought to determine whether ABT is able to inhibit HIV-1 strains resistant to T20. Therefore, we generated a panel of HIV-1_NL4-3_ Env-based pseudoviruses carrying single or double mutations that are frequently emerged in T20- treated AIDS patients and approved to confer T20-resistance (Table 3). Different from the majority of HIV-1 strains that carry a “GIV” motif, HIV-1_NL4-3_ inherits a “DIV” motif that is well known to confer T20-resistance. Therefore, we calculated the resistance fold-changes by using both the wild-type and D36G mutant as reference viruses (Table 3). As expected, D36G mutant was more sensitive to T20 inhibition (8.3-fold) while not significantly changed to C34 and ABT. Impressively, single or double mutations in the gp41 could result in high level resistance to T20 and cross-resistance to C34. In contrast, ABT could efficiently inhibit these variants. For example, V38A or V38M single mutations and V38A/N42T double mutations conferred high-fold resistance changes to T20 and C34 but much mild to ABT. We also chose two HIV-1_NL4-3_ molecular clones carrying L33S or I37V/V38T to evaluate ABT and T20. The results showed that ABT was also highly effective on these two infectious viruses. In a sharp contrast, T20 had no any inhibitory activity at a concentration up to 750 nM. Therefore, the present data highlight that ABT has a potent and broad anti-HIV activity, not only for currently worldwide circulating subtypes but also for the induced variants that are resistant to the first and second generations of HIV-1 fusion inhibitors.

**Table 3:** Inhibition of ABT on T20-resistant HIV-1 variants^a^.

HIV-1_NL4-3_ b	T20		C34		ABT		AZT	
	IC_50_ (nM)	Fold change^c^	IC_50_ (nM)	Fold change	IC_50_ (nM)	Fold change	IC_50_ (nM)	Fold change
WT	62.5±11.9	1.0 (8.3)	2.0±0.6	1.0 (1.8)	1.9±0.4	1.0 (0.6)	106.7±23.2	1.0 (1.8)
D36G	7.5±1.3	0.1 (1.0)	1.1±0.1	0.6 (1.0)	3.3±0.8	1.7 (1.0)	59.4±5.9	0.6 (1.0)
I37T	300.2±55.1	4.8 (40.0)	20.5±1.7	10.3 (18.6)	7.6±1.0	4.0 (2.3)	93.5±13.5	0.9 (1.6)
V38A	1261.0±182.3	20.2 (168.1)	47.2±10.8	23.6 (42.9)	8.5±1.2	4.5 (2.6)	42.3±9.0	0.4 (0.7)
V38M	645.6±57.7	10.3 (86.1)	16.5±4.2	8.3 (15.0)	8.7±1.4	4.5 (2.6)	47.8±3.7	0.5 (0.8)
Q40H	731.4±35.2	11.7 (97.5)	39.9±5.3	20.0 (36.3)	9.7±2.0	6.5 (2.9)	33.2±7.2	0.3 (0.6)
N43K	262.3±44.4	4.2 (35.0)	26.3±3.5	13.2 (23.9)	5.9±0.9	3.1 (1.8)	153.3±7.4	1.4 (2.6)
D36S/V38M	322.5±60.3	5.2 (43.0)	11.3±1.6	5.7 (10.3)	10.3±0.9	5.4 (3.1)	40.3±8.6	0.4 (0.7)
I37T/N43K	>3,000.0	>48.0 (>400.0)	261.8±47.4	131.9 (238.0)	32.4±5.9	17.1 (9.8)	138.9±14.9	1.3 (2.3)
V38A/N42T	>3,000.0	>48.0 (>400.0)	444.3±133	222.2 (403.9)	7.8±1.5	4.1 (2.4)	38.1±3.7	0.4 (0.6)
L33S	>750.0	NA	ND	NA	2.7±0.3	NA	ND	NA
I37V/V38T	>750.0	NA	ND	NA	4.1±0.8	NA	ND	NA

a The data were derived from the results of three independent experiments and are expressed as means ± standard deviations.

b HIV-1NL4-3-based pseudoviruses were constructed and used in the single-cycle infection assays except that L33S and I37V/V38T are infectious molecular clones of HIV-1NL4-3.

c Both wild-type (WT) and D36G were used as reference viruses to calculate the resistance fold-changes.Shown in parentheses are based on the D36G as a reference.

## Discussion

ABT is a newly developed MPA-modified anti-HIV peptide that can irreversibly conjugate to the serum albumin and extend its half-life [25]. The previous studies had shown its *in vivo* longevity and anti-HIV activity and pushed it in a Phase I clinical trial in China. In the present study, we have finely characterized ABT by using several approaches. Firstly, we analyzed its biophysical properties by CD and ITC analyses. It is well known that the CD spectra can reveal the presence of stable α-helical structures of the NHR-CHR helical bundle that is thought to be mechanistically and thermodynamically correlated with HIV-1 fusion [26], [31]. The CD spectra typically at 222 nm indicated the inter-helical interaction of ABT and N36, a NHR-derived peptide containing the ABT target sequence. The α-helicity and thermostability of ABT/N36 mixture were higher than that of C34/N36 mixture, a core structure of fusion-active gp41 [1]. The high-affinity interaction of ABT and N36 was further confirmed by ITC experiments. These results suggested that the chemically modified peptide fusion inhibitor ABT could maintain even improve its normal structure and function. However, how the ABT/N36 interaction displayed higher α-helicity than the C34/N36 complex need to be further characterized. One can speculate that mutated residues or MPA molecules within ABT may determine the difference.

The mechanism of action of HIV-1 peptide inhibitors derived from the NHR and CHR has been considered to be a dominant-negative manner, in which the exogenous peptides block the formation of 6-HB structure through the competitive binding [6], [10]. In our previous studies, we developed an ELISA-based system to test whether the peptide-based or small molecule-based fusion inhibitors block the 6-HB formation, in which the mAb NC-1 specific for the 6-HB was used as a capture and the biotinylated-C34 was used for signal detection [26], [32]. With this convenient method we proved that ABT could efficiently block the 6-HB formation. Together with the CD and ITC data, our studies demonstrated that ABT acts like many other peptides through a mechanism of 6-HB inhibition. Concomitant to its mechanism of interacting with the NHR target and disrupting 6-HB, ABT had potent inhibitory activity on HIV-1 Env-mediated cell-cell membrane fusion and virus entry. We also demonstrated that ABT could stably exert its strong anti-HIV-1 activity in freshly prepared human sera, suggesting its *in vivo* stability in the same manner that T20 does. Actually, the previous studies have indicated that the anti-HIV activity of peptide-based fusion inhibitors is not significantly influenced by serum components [19], [33].

One of the obstacles to treatment of the HIV-1 is its extremely high genetic variability, which results in many subtypes and CRFs. According to the epidemiological data, HIV-1 subtypes and CRFs are very unevenly distributed throughout the world, with the most widespread being subtypes A, B and C [27]–[28]. Subtype A viruses predominate in west and central Africa, and subtype B is the most common form in Europe, the Americas, Japan, Thailand, and Australia; Subtype C is the dominant form in southern and eastern Africa, India, and Nepal and is responsible for about half of worldwide infections. In China, CRF07_BC, CRF01_AE and subtype B′ have caused the worst HIV-1 epidemics [29]–[30]. Because some *in vitro* and *in vivo* observations suggest that the various subtypes may respond differently to certain antiretroviral drugs [34]–[38], we have evaluated ABT for its anti-HIV spectrum by using two large panels of HIV-1 pseudoviruses with their Envs corresponding to the above diverse variants. Sequence analyses indicate that these viral Envs exhibit high genetic variations, not only in gp120 but also in the NHR and CHR of gp41. Furthermore, we have also evaluated ABT for its inhibitory activity on a panel of HIV-1 variants that are highly resistant to T20. The presented results have demonstrated that ABT has potent and broad antiviral activity against diverse HIV-1 variants, suggesting its relatively higher genetic barrier for drug-resistance.

Human serum albumin (HSA) constitutes about half of the blood serum protein and is the most abundant protein in human plasma. Due to its serum half-life of approximately 20 days and well distribution in different tissues, it has been used as a drug carrier for small molecule, peptides and protein-based therapeutics and shows excellent safety profiles and therapeutic efficacy [38]–[43]. ABT was designed to be a long-lasting peptide inhibitor that can automatically link the serum albumin and exert *in vivo* anti-HIV activity after injection [25]. The efficient *in vivo* protein binding capacity and the significantly prolonged *in vivo* half-life of ABT were verified in the previous studies. Its potent *in vivo* anti-HIV activity was also demonstrated by a SCID-hu Thy/Liv mouse model [25]. Being a molecular mass of 67 Kd, one may speculate that HSA can impose a steric hindrance to the accessibility of the NHR target during the pre-hairpin state of gp41 [44], but it is not the case for HSA-conjugated CHR peptides. It was observed by *in vitro* experiments that HSA-conjugated ABT had a similar anti-HIV activity with the free ABT peptide (data not shown). Our data also showed that ABT-HSA conjugates could efficiently block 6-HB formation and membrane fusion, explaining how ABT can act well after its conjugation to the serum albumin. Recently, several groups have validated the accessibility of NHR pre-hairpin intermediate by large molecules, such as neutralizing antibodies and protein-based inhibitors [45]–[46].

Recently, HSA was conjugated to the N- or C-termini of C34 and T20 [24]. It showed that the designed bioconjugates, such as PC-1505 were essentially equipotent to the parent peptides *in vitro* and possessed significantly improved pharmacokinetic profiles and potent anti-HIV activity *in vivo*. Differently, ABT peptide was modified by a MPA molecule in the middle region of its body (Fig. 1) and could be automatically conjugated *in vivo* upon injection [25]. We are wondering how can ABT, after its tight attachment to HSA, keep its flexibility to interact with the NHR region and exerts its inhibition on 6-HB? In other words, positioning of ABT through its middle to a large cargo does not affect its NHR binding to form a stable 6-HB structure. We are on the way to determine the crystal structure of ABT-HSA conjugate in the presence and absence of a NHR peptide, and hopefully, this study will deliver more detailed information on the antiviral mechanism of ABT. It should be discussed that the albumin molecule may also play an active participatory role rather than only serving as a protein cargo. In fact, the previous studies demonstrated that the chemically modified albumins are potent HIV entry inhibitors [47]–[50]. Furthermore, Jacobs et al [51] reported that the MPA-modified C34 could serve as a covalent inhibitor targeting an intermediate conformation of the gp41 during the fusion. It is possible the permanent specific attachment of the covalent inhibitor can improve the pharmacokinetics of administration *in vivo* and thereby improve the long-term sustainability of peptide inhibitor therapy. It will be interesting to explore whether ABT has such effect.

In summary, the significance of the present study is threefold. First, it shows the α-helical and thermostable properties of MPA-modified peptide fusion inhibitor. Second, it reveals that ABT acts through a mechanism of blocking fusion-active 6-HB formation and membrane fusion. Third, it demonstrates that ABT possesses potent and broad inhibitory activity against diverse HIV-1 subtypes and variants. Therefore, our data indicate that the chemically modified anti-HIV peptide ABT can serve as an ideal HIV fusion inhibitor that may possess long-lasting activity. The information presented by this study is critical for guiding ABT for its ongoing clinical development.

## Materials and Methods

### Peptide synthesis

Peptides for ABT, T20, C34, and N36 were synthesized on a standard solid-phase support, purified by reversed-phase high pressure liquid chromatography, and verified for purity >95% by mass spectrometry and amino acid composition. The side chain of 13^th^ lysine residue of the peptide ABT was protected by allyloxycarbonyl (Aloc), which allows the specific deprotection and addition of the linker molecule and MPA. Concentrations of the peptides were determined by UV absorbance and a theoretically calculated molar-extinction coefficient ∈(280 nm) of 5500 M^−1^ cm^−1^ and 1490 M^−1^ cm^−1^ based on the number of tryptophan and tyrosine residues (all the peptides tested contain Trp and/or Tyr), respectively.

### HIV-1 Env-expressing plasmids

A panel of plasmids encoding HIV-1 Envs were obtained through the AIDS Research and Reference Reagent Program (ARRRP), Division of AIDS, NIAID, NIH, including subtype A Env clones pSVIII-92RW020.5 and 92UG037.8 from Dr. B.H. Hahn; subtype B Env clones SC422661.8, TRO.11 and AC10.0.29 from Drs. D. Montefiori, F. Gao and M. Li, pRHPA4259.7 and pREJO4541.67 from Drs. B.H. Hahn and J.F. Salazar-Gonzalez, and pCAAN5342.A2 from Drs. B.H. Hahn and D.L. Kothe; subtype C Env clones Du172.17 and Du422.1 from Drs. D. Montefiori, F. Gao, S. Abdool Karim and G. Ramjee, CAP45.2.00.G3 from Drs. L. Morris, K. Mlisana and D. Montefiori, ZM197M.PB7 from Drs. B.H. Hahn, Y. Li and J.F. Salazar-Gonzalez, ZM109F.PB4 from Drs. E. Hunter and C. Derdeyn. Six CRF07_BC Env clones (CH064.20, CH070.1, CH091.9, CH110.2, CH119.10 and CH120.6) were generous gifts by Dr. Y. Shao in the Chinese Center for Disease Control and Prevention, Beijing, China. A panel of Env clones derived from subtype B′ (B01, B02, B04, and 43-22), CRF01_AE (SHX335.24, YN192.31, AE01, AE03, GX2010.36, GX11.13, GX2010.36H, BJ5.11), and CRF07_BC (BC02, BC03, BC05, BC07, BC14, SC19-15, BJ22-5, YN148R-9, XJ50-6, HB5-3) were kindly provided by Dr. Y. Wang from the National Institute for the Control of Pharmaceutical and Biological Products, Beijing, China.

### Cell lines

TZM-bl cells were contributed by J.C. Kappes and X. Wu through the ARRRP, Division of AIDS, NIAID, NIH. This reporter cell line stably expresses high levels of CD4, CCR5, and CXCR4 and contains Tat-responsive reporter genes for firefly luciferase and β-galactosidase under the control of an HIV-1 long terminal repeat promoter [52]. HL2/3 cells stably expressing HIV Gag, Env, Tat, Rev, and Nef proteins were obtained through the ARRRP, NIH from Drs. B. Felber and G. Pavlakis. 293T cells were obtained from the American Type Culture Collection (ATCC, Manassas, VA). All cell lines were maintained in Dulbecco's modified Eagle's medium (DMEM) growth medium (Gibco/Invitrogen) containing 10% heat-inactivated fetal bovine serum (FBS) at 37°C in humidified air containing 5% CO_2_. Cells were harvested using trypsin/EDTA solution (Gibco/Invitrogen).

### Circular dichroism (CD) spectroscopy

CD spectroscopy was performed as previously described [15], [26]. Briefly, a C-peptide (ABT, T20 or C34) was incubated with the equal molar concentration of NHR-derived peptide N36 at 37°C for 30 min. The final peptide concentration was 10 µM in PBS buffer (pH 7.2). The isolated N- and C-peptides were also tested. The CD spectra of these individual peptides and peptide mixtures were acquired on MOS-450 spectropolarimeter (Bio-Logic, France) using a 1 nm bandwidth with a 1 nm step resolution from 190 to 260 nm at room temperature. The spectra were corrected by subtraction of a blank corresponding to the solvent. Data were averaged over three accumulations. The α-helical content was calculated from the CD signal by dividing the mean residue ellipticity [θ] at 222 nm by the value expected for 100% helix formation (−33,000 degrees.cm^2^.dmol^−1^) as described previously [53]–[54]. The thermal denaturation experiment was performed by monitoring the change in ellipticity [θ] at 222 nm at the increasing temperature (20–90°C) using temperature controller. The temperature was increased at a rate of 1°C per min; data were acquired at a 2 nm bandwidth at 222 nm at a frequency of 0.25 Hz. The melting curve was smoothened, and the midpoint of the thermal unfolding transition (*Tm*) values were taken as the maximum of the derivative d[θ]_222_/dT. *Tm* value was detected at protein concentration of 1 µM in PBS buffer.

### Isothermal Titration Calorimetry (ITC) assay

ITC assay was performed using a ITC_200_ Microcalorimeter instrument (MicroCal, USA) as described previously [55]. In brief, 2 mM N36 dissolved in ddH_2_0 was injected into the chamber containing 200 µM ABT, C34 or T20, respectively. The experiments were carried out at 25°C. The time between injections was 240 seconds and the stirring speed was 500 rpm. The heats of dilution were determined in control experiments by injecting N36 into ddH_2_0 and subtracted from the heats produced in the corresponding peptide-peptide binding experiments. Data acquisition and analysis were performed using MicroCal Origin software (version 7.0).

### Inhibition of 6-HB formation by peptides

A monoclonal antibody specific for the gp41 6-HB (NC-1) was obtained from Dr. Shibo Jiang through the ARRRP, Division of AIDS, NIAID, NIH. Inhibitory activity of ABT, T20 and C34 on the 6-HB formation was measured by a modified ELISA-based method as previously described [26], [32]. Briefly, a 96-well polystyrene plate (Costar, Corning Inc., Corning, NY) was coated with NC-1 (2 µg/ml in 0.1 M Tris, pH 8.8). A tested peptide (ABT, C34 or T20) at graded concentrations was mixed with C34-biotin (0.1 µM) and incubated with N36 (0.1 µM) at room temperature for 30 min. The mixture was then added to the NC-1-coated plate, followed by incubation at room temperature for 30 min and washing with a washing buffer (PBS containing 0.1% Tween 20) three times. Then streptavidin-labeled horseradish peroxidase (Invitrogen) and the substrate 3,3,5,5- tetramethylbenzidine (Sigma) were added sequentially. Absorbance at 450 nm (*A*450) was measured using an ELISA reader (Bio-Rad).

### Cell-cell fusion assay

Cell fusion was monitored using a reporter gene assay based on activation of HIV LTR-driven luciferase cassette in TZM-bl (Target) cells by HIV-1 tat from the HL2/3 (Effector) cells. Briefly, the TZM-bl cells were plated in 96-well clusters (2×10^4^ per well) and incubated at 37°C overnight. The target cells were co-cultured with HL2/3 cells (6×10^4^/well) for 6 hours at 37°C in the presence or absence of a tested peptide (ABT, T20 or C34) at graded concentrations. Luciferase activity was measured using luciferase assay regents (Promega, Medison, WI) and a Luminescence Counter (Promega) according to the manufacture's instructions. Background luminescence in TZM-bl cells was determined without addition of HL2/3 cells. The percent inhibition of fusion by the peptides and 50% inhibitory of fusion concentration (IC_50_) values were calculated as previously described [15].

### Site-directed mutagenesis

A panel of HIV-1_NL4-3_ mutants resistant to T20 was generated as described previously [56]. The mutations were created using double-stranded DNA templates and selection of mutants with *DpnI*. For each mutation, the two primers contained the desired mutation and occupied the same starting and ending positions on opposite strands of the plasmid DNA. DNA synthesis was carried out by PCR in a 50-µL reaction volume using 1 µL of denatured plasmid template (1 ng/µL), 50 pmol of upper and lower primers and 5 U of high-fidelity thermostable polymerase PrimeStar (Takara, Dalian, China). PCR amplification was carried out for one cycle of denaturation at 98°C for 5 min, then 18 cycles of 98°C for 15 s and 68°C for 15 min, followed a final extension at 72°C for 10 min. The amplicons were treated with the restriction enzyme *DpnI* for 1 h at 37°C. *DpnI*-resistant molecules, which were rich in the desired mutants, were recovered by transforming *Escherichia coli* strain-DH5α to antibiotic resistance. The successful mutations were confirmed by sequencing.

### Inhibition of HIV-1 single-cycle infection

HIV-1 pseudoviruses were generated as described previously [32], [55]–[56]. Briefly, 293T cells (5×10^6^ cells in 15 ml of growth medium in a T-75 culture flask) were cotransfected with 10 µg of an Env-expressing plasmid and 20 µg of a backbone plasmid pSG3deltaEnv that encodes Env-defective, luciferase-expressing HIV-1 genome using Lipofectamine 2000 reagent (Invitrogen). Pseudovirus-containing culture supernatants were harvested 48 hours after transfection and filtered by 0.45-mm pore size, and stored at −80°C in 1-ml aliquots until use. The 50% tissue culture infectious dose (TCID_50_) of a single thawed aliquot of each pseudovirus batch was determined in TZM-bl cells. The antiviral activity of ABT or T20 was determined using TZM-b1 cells as described previously [21]. Briefly, the peptides were prepared with ten series dilutions in a 3-fold stepwise manner and mixed with 100 TCID_50_ viruses and incubated 1 hour at room temperature. The mixture was added to TZM-bl cells (10^4^/well) and incubation at 37°C for 48 hours. Luciferase activity was measured using luciferase assay regents (Promega, Medison, WI) and a Luminescence Counter (Promega) according to the manufacture's instructions. The percent inhibition by the peptides and 50% inhibitory concentration (IC_50_) values were calculated as previously described [21].

### Inhibition of infectious HIV-1_NL4-3_ variants

HIV-1 molecular clone NL4-3 carrying L33S or I37V/V38T mutation [57]–[58] was kindly provided by Dr. Frank Kirchhoff from the Institute of Virology, the University of Ulm, Ulm, Germany. The mutant viruses were generated by transient transfection of NL4-3 plasmids into 293T cells. The virus stocks were harvested 48 hours post-transfection and quantified for TCID_50_. Inhibition of the peptides (ABT and T20) on the NL4-3 mutants was performed as described for pseudoviruses. In brief, 100 TCID_50_ viruses were used to infect TZM-bl cells in the presence or absence of serially diluted peptides. Two days post-infection, the cells were harvested and lysed in reporter lysis buffer, and the luciferase activity was measured. The IC_50_ was calculated as described above using GraphPad Prism software (GraphPad Prism software Inc., San Diego, CA).

## References

[1] Chan DC, Fass D, Berger JM, Kim PS (1997). Core structure of gp41 from the HIV envelope glycoprotein.. Cell.

[2] Tan K, Liu J, Wang J, Shen S, Lu M (1997). Atomic structure of a thermostable subdomain of HIV-1 gp41.. Proc Natl Acad Sci U S A.

[3] Weissenhorn W, Dessen A, Harrison SC, Skehel JJ, Wiley DC (1997). Atomic structure of the ectodomain from HIV-1 gp41.. Nature.

[4] Buzon V, Natrajan G, Schibli D, Campelo F, Kozlov MM (2010). Crystal structure of HIV-1 gp41 including both fusion peptide and membrane proximal external regions.. PLoS Pathog.

[5] Berkhout B, Sanders RW (2011). Molecular strategies to design an escape-proof antiviral therapy.. Antiviral Res.

[6] Eggink D, Berkhout B, Sanders RW (2010). Inhibition of HIV-1 by fusion inhibitors.. Curr Pharm Des.

[7] Steffen I, Pohlmann S (2010). Peptide-based inhibitors of the HIV envelope protein and other class I viral fusion proteins.. Curr Pharm Des.

[8] Jiang S, Lin K, Strick N, Neurath AR (1993). HIV-1 inhibition by a peptide.. Nature.

[9] Wild CT, Shugars DC, Greenwell TK, McDanal CB, Matthews TJ (1994). Peptides corresponding to a predictive alpha-helical domain of human immunodeficiency virus type 1 gp41 are potent inhibitors of virus infection.. Proc Natl Acad Sci U S A.

[10] Chan DC, Kim PS (1998). HIV entry and its inhibition.. Cell.

[11] Kilby JM, Hopkins S, Venetta TM, DiMassimo B, Cloud GA (1998). Potent suppression of HIV-1 replication in humans by T-20, a peptide inhibitor of gp41-mediated virus entry.. Nat Med.

[12] Lalezari JP, Henry K, O'Hearn M, Montaner JS, Piliero PJ (2003). Enfuvirtide, an HIV-1 fusion inhibitor, for drug-resistant HIV infection in North and South America.. N Engl J Med.

[13] Patel IH, Zhang X, Nieforth K, Salgo M, Buss N (2005). Pharmacokinetics, pharmacodynamics and drug interaction potential of enfuvirtide.. Clin Pharmacokinet.

[14] Eggink D, Baldwin CE, Deng Y, Langedijk JP, Lu M (2008). Selection of T1249-resistant human immunodeficiency virus type 1 variants.. J Virol.

[15] He Y, Cheng J, Lu H, Li J, Hu J (2008). Potent HIV fusion inhibitors against Enfuvirtide-resistant HIV-1 strains.. Proc Natl Acad Sci U S A.

[16] He Y, Xiao Y, Song H, Liang Q, Ju D (2008). Design and evaluation of sifuvirtide, a novel HIV-1 fusion inhibitor.. J Biol Chem.

[17] Dwyer JJ, Wilson KL, Davison DK, Freel SA, Seedorff JE (2007). Design of helical, oligomeric HIV-1 fusion inhibitor peptides with potent activity against enfuvirtide-resistant virus.. Proc Natl Acad Sci U S A.

[18] Naider F, Anglister J (2009). Peptides in the treatment of AIDS.. Curr Opin Struct Biol.

[19] Nishikawa H, Nakamura S, Kodama E, Ito S, Kajiwara K (2009). Electrostatically constrained alpha-helical peptide inhibits replication of HIV-1 resistant to enfuvirtide.. Int J Biochem Cell Biol.

[20] Eggink D, Langedijk JP, Bonvin AM, Deng Y, Lu M (2009). Detailed mechanistic insights into HIV-1 sensitivity to three generations of fusion inhibitors.. J Biol Chem.

[21] Yao X, Chong H, Zhang C, Waltersperger S, Wang M (2012). Broad antiviral activity and crystal structure of HIV-1 fusion inhibitor Sifuvirtide.. J Biol Chem.

[22] Wexler-Cohen Y, Shai Y (2007). Demonstrating the C-terminal boundary of the HIV 1 fusion conformation in a dynamic ongoing fusion process and implication for fusion inhibition.. FASEB J.

[23] Ingallinella P, Bianchi E, Ladwa NA, Wang YJ, Hrin R (2009). Addition of a cholesterol group to an HIV-1 peptide fusion inhibitor dramatically increases its antiviral potency.. Proc Natl Acad Sci U S A.

[24] Stoddart CA, Nault G, Galkina SA, Thibaudeau K, Bakis P (2008). Albumin-conjugated C34 peptide HIV-1 fusion inhibitor: equipotent to C34 and T-20 in vitro with sustained activity in SCID-hu Thy/Liv mice.. J Biol Chem.

[25] Xie D, Yao C, Wang L, Min W, Xu J (2010). An albumin-conjugated peptide exhibits potent anti-HIV activity and long in vivo half-life.. Antimicrob Agents Chemother.

[26] He Y, Cheng J, Li J, Qi Z, Lu H (2008). Identification of a critical motif for the human immunodeficiency virus type 1 (HIV-1) gp41 core structure: implications for designing novel anti-HIV fusion inhibitors.. J Virol.

[27] McCutchan FE (2000). Understanding the genetic diversity of HIV-1.. Aids.

[28] Taylor BS, Hammer SM (2008). The challenge of HIV-1 subtype diversity.. N Engl J Med.

[29] Liao L, Xing H, Shang H, Li J, Zhong P (2010). The prevalence of transmitted antiretroviral drug resistance in treatment-naive HIV-infected individuals in China.. J Acquir Immune Defic Syndr.

[30] Lu L, Jia M, Ma Y, Yang L, Chen Z (2008). The changing face of HIV in China.. Nature.

[31] Eckert DM, Kim PS (2001). Mechanisms of viral membrane fusion and its inhibition.. Annu Rev Biochem.

[32] He Y, Liu S, Li J, Lu H, Qi Z (2008). Conserved salt bridge between the N- and C-terminal heptad repeat regions of the human immunodeficiency virus type 1 gp41 core structure is critical for virus entry and inhibition.. J Virol.

[33] Naito T, Izumi K, Kodama E, Sakagami Y, Kajiwara K (2009). SC29EK, a peptide fusion inhibitor with enhanced alpha-helicity, inhibits replication of human immunodeficiency virus type 1 mutants resistant to enfuvirtide.. Antimicrob Agents Chemother.

[34] Palmer S, Alaeus A, Albert J, Cox S (1998). Drug susceptibility of subtypes A,B,C,D, and E human immunodeficiency virus type 1 primary isolates.. AIDS Res Hum Retroviruses.

[35] Loemba H, Brenner B, Parniak MA, Ma'ayan S, Spira B (2002). Genetic divergence of human immunodeficiency virus type 1 Ethiopian clade C reverse transcriptase (RT) and rapid development of resistance against nonnucleoside inhibitors of RT.. Antimicrob Agents Chemother.

[36] Brenner B, Turner D, Oliveira M, Moisi D, Detorio M (2003). A V106M mutation in HIV-1 clade C viruses exposed to efavirenz confers cross-resistance to non-nucleoside reverse transcriptase inhibitors.. Aids.

[37] Grossman Z, Istomin V, Averbuch D, Lorber M, Risenberg K (2004). Genetic variation at NNRTI resistance-associated positions in patients infected with HIV-1 subtype C.. Aids.

[38] Jette L, Leger R, Thibaudeau K, Benquet C, Robitaille M (2005). Human growth hormone-releasing factor (hGRF)1-29-albumin bioconjugates activate the GRF receptor on the anterior pituitary in rats: identification of CJC-1295 as a long-lasting GRF analog.. Endocrinology.

[39] Hartung G, Stehle G, Sinn H, Wunder A, Schrenk HH (1999). Phase I trial of methotrexate-albumin in a weekly intravenous bolus regimen in cancer patients. Phase I Study Group of the Association for Medical Oncology of the German Cancer Society.. Clin Cancer Res.

[40] Cruz LJ, Iglesias E, Aguilar JC, Quintana D, Garay HE (2001). Study of different coupling agents in the conjugation of a V3-based synthetic MAP to carrier proteins.. J Pept Sci.

[41] Balan V, Nelson DR, Sulkowski MS, Everson GT, Lambiase LR (2006). A Phase I/II study evaluating escalating doses of recombinant human albumin-interferon-alpha fusion protein in chronic hepatitis C patients who have failed previous interferon-alpha-based therapy.. Antivir Ther.

[42] Abu Ajaj K, Graeser R, Fichtner I, Kratz F (2009). In vitro and in vivo study of an albumin-binding prodrug of doxorubicin that is cleaved by cathepsin B.. Cancer Chemother Pharmacol.

[43] Esmaeili F, Dinarvand R, Ghahremani MH, Amini M, Rouhani H (2009). Docetaxel-albumin conjugates: preparation, in vitro evaluation and biodistribution studies.. J Pharm Sci.

[44] Hamburger AE, Kim S, Welch BD, Kay MS (2005). Steric accessibility of the HIV-1 gp41 N-trimer region.. J Biol Chem.

[45] Sabin C, Corti D, Buzon V, Seaman MS, Lutje Hulsik D (2010). Crystal structure and size-dependent neutralization properties of HK20, a human monoclonal antibody binding to the highly conserved heptad repeat 1 of gp41.. PLoS Pathog.

[46] Miller MD, Geleziunas R, Bianchi E, Lennard S, Hrin R (2005). A human monoclonal antibody neutralizes diverse HIV-1 isolates by binding a critical gp41 epitope.. Proc Natl Acad Sci U S A.

[47] Takami M, Sone T, Mizumoto K, Kino K, Tsunoo H (1992). Maleylated human serum albumin inhibits HIV-1 infection in vitro.. Biochim Biophys Acta.

[48] Jansen RW, Schols D, Pauwels R, De Clercq E, Meijer DK (1993). Novel, negatively charged, human serum albumins display potent and selective in vitro anti-human immunodeficiency virus type 1 activity.. Mol Pharmacol.

[49] Swart PJ, Beljaars E, Smit C, Pasma A, Schuitemaker H (1996). Comparative pharmacokinetic, immunologic and hematologic studies on the anti-HIV-1/2 compounds aconitylated and succinylated HSA.. J Drug Target.

[50] Groenink M, Swart PJ, Broersen S, Kuipers M, Meijer DK (1997). Potent inhibition of replication of primary HIV type 1 isolates in peripheral blood lymphocytes by negatively charged human serum albumins.. AIDS Res Hum Retroviruses.

[51] Jacobs A, Quraishi O, Huang X, Bousquet-Gagnon N, Nault G (2007). A covalent inhibitor targeting an intermediate conformation of the fusogenic subunit of the HIV-1 envelope complex.. J Biol Chem.

[52] Derdeyn CA, Decker JM, Sfakianos JN, Wu X, O'Brien WA (2000). Sensitivity of human immunodeficiency virus type 1 to the fusion inhibitor T-20 is modulated by coreceptor specificity defined by the V3 loop of gp120.. J Virol.

[53] Chen YH, Yang JT, Chau KH (1974). Determination of the helix and beta form of proteins in aqueous solution by circular dichroism.. Biochemistry.

[54] Shu W, Liu J, Ji H, Radigen L, Jiang S (2000). Helical interactions in the HIV-1 gp41 core reveal structural basis for the inhibitory activity of gp41 peptides.. Biochemistry.

[55] He Y, Liu S, Jing W, Lu H, Cai D (2007). Conserved residue Lys574 in the cavity of HIV-1 Gp41 coiled-coil domain is critical for six-helix bundle stability and virus entry.. J Biol Chem.

[56] Chong H, Xu S, Zhang C, Nie J, Wang Y (2009). Mutation L33M in the HR1 region of HIV-1 gp41 may play a role in T20 resistance.. J Clin Virol.

[57] Chinnadurai R, Munch J, Kirchhoff F (2005). Effect of naturally-occurring gp41 HR1 variations on susceptibility of HIV-1 to fusion inhibitors.. Aids.

[58] Chinnadurai R, Rajan D, Munch J, Kirchhoff F (2007). Human immunodeficiency virus type 1 variants resistant to first- and second-version fusion inhibitors and cytopathic in ex vivo human lymphoid tissue.. J Virol.

